# A Drug Utilization Evaluation Study of Amphotericin B in Neutropenic Patients in a Teaching Hospital in Iran

**Published:** 2012

**Authors:** Maria Tavakoli-Ardakani, Azadeh Eshraghi, Azita Hajhossein Talasaz, Jamshid Salamzadeh

**Affiliations:** a *Department of Clinical Pharmacy, School of Pharmacy, Shahid Beheshti University of Medical Sciences.*; b *Pharmaceutical Science Research Center, Shahid Beheshti University of Medical Sciences.*; c *Department of Clinical Pharmacy, Faculty of Pharmacy, Tehran University of Medical Sciences.*

**Keywords:** Amphotericin B, Drug Utilization Evaluation (DUE), Febrile neutropenia

## Abstract

Drug Utilization Evaluation (DUE) studies facilitate assessing the appropriateness and rational use of medications.The goal of the present study was to evaluate Amphotericin B usage in neutropenic patients. A prospective DUE study was performed in Hematology-Oncology and Stem Cell Transplantation wards at Taleghani hospital for one-year. National comprehensive cancer network, clinical practice guidelines in oncology, American Hospital Formulary Service and other relevant medical practice and up-to dated articles were used to evaluate whether Amphotericin B is properly used according to the guidelines. All data collected by a pharmacist in daily review using information of physician and nursing records as well as laboratory findings. During the one-year study, 35 patients receiving amphotericin B were evaluated. 29 patients (82.9%) received amphotericin B due to neutropenia and fever and 6 patients had confirmed fungal infections. All of the injectable solutions of amphotericin B were appropriately prepared for intravenous infusion. In addition, for all patients, ordering (indication) of the study drug was in accordance with the guidelines. Twenty-five (71.4%) patients received an appropriate dose according to the guidelines. Duration of treatment was properly selected in 21 (60%) patients. Twenty-two (62.8%) patients developed hypokalemia as the most frequent adverse drug event. Although, preparation and indication of amphotericin B was in compliance with the current guidelines, dosage and duration of treatment were considered to be incoherent with the designed protocol used in this study. We conclude more attention should be paid to dosage and duration of treatment with amphotericin B in order to optimize its administration.

## Introduction

Fungal pathogens are considered as causative organisms of infections in patients with immunesuppression, attributable to neutropenia. Most of the common fungal infections in neutropenic patients are caused by *Candida *and *Aspergillus *species. Antifungal medications are useful in eradicating fungal infections in subjects with hematologic malignancies precipitated as a consequence of prolonged immunosuppressive therapy especially in those with neutropenia (1). Bacterial infection occurring most often early in febrile neutropenic subjects along with fungal late-onset infections, lead to a high rate of morbidity and mortality among neutropenic patients undergoing chemotherapy. In 1980s empiric therapy with Amphotericin B was considered in severe fungal infections in neutropenic patients. Until recently, it is administered as a standard strategy in neutropenic patients with fever unresponsive to broad spectrum antibiotics (2, 3). Amphotericin B desoxycholate, or conventional amphotericin B, is an effective antifungal treatment in serious fungal infections including *Candida *and *Aspergillus *species, *Coccidio idesimmitis, Histoplasmaca psulatum, Blastomyces dermatitidis, Cryptococcus neoformans *and *Sporothrix schenckii*. It has activity against fungal infections in immunocompromised patients who are neutropenic (4). Adverse effects consisting of pyrexia, rigors, phlebitis, myalgias, malaise, nausea, vomiting, hepatotoxicity and hypokalemia are common with amphotericin B. Amphotericin B administration may result in nephrotoxicity that is dose dependent. Damageto renal tubules as a result of amphotericin administration can lead to acute kidney injury (AKI) (5). AKI was reported in 49% to 65% of patients receiving amphotericin B. 

Although the effectiveness of DUE programs has yet to be established, drug utilization evaluation (DUE) studies are still used to identify variability in drug use as well as to support interventions that will improve patient outcomes (6).

Due to the frequency of renal toxicity and other side effects associated with amphotericin B (7), we conducted a drug use evaluation (DUE) study to determine whether its administration for empirical antifungal therapy in neutropenic patients is according to the approved guidelines. 

## Experimental

*Patients and methods*

This prospective drug utilization study was carried out at Taleghani hospital affiliated to Shahid Beheshti Medical University, Tehran, Iran between 2008 and 2009 on 35 patients who were hospitalized in bone marrow transplantation (BMT), and oncology -hematology wards. A questionnaire was prepared based on American hospital formulary service (AHFS) and other reliable articles. This questionnaire included patients’ demographic data such as age, sex, drug history, medical background, and laboratory tests before treatment’s initiation. Blood urea nitrogen (BUN), creatinine, potassium level, magnesium level, and complete blood count (CBC) were the documented laboratory tests. The type of infection, patients’ vital signs during infusion, any adverse drug reactions in the treatment course, and culture results were recorded in the prepared questionnaire. Also a predefined protocol based on national comprehensive cancer network (NCCN) criteria was utilized to assess the appropriateness of the neutropenic cancer patients’ management (8). All patient- specific data were extracted by a pharmacist observation and daily review using information of physician orders, nursing records, laboratory findings and patient’s interview. Patients were followed up until the end of treatment course. Collected data entered the computer softwares of Excel 2007 and SPSS (version 17) for descriptive analysis. 

## Results

During one year period, 35 neutropenic patients including 20 males (57.14%) and 15 females (42.86%) with mean ± SD age of 41.88 ± 12.58 years (range 19 to 65 years) were evaluated. Three (8.57%) patients were from the BMT ward. Six patients had proven fungal infections including 2 (5.71%) mucormycosis, 3 (8.57%) aspergillosis and 1(2.86%) candidasis. Distribution of patients based on their underlying disease was shown in Table 1. 

**Table 1:** Frequency and percentages of underlying disease in included patients (n = 35).

**Underlying disease**	**Type of disease**	**Frequency (%)**
**Solid tumor**	**Neck tumor**	**1 (2.9)**
Blood disease	Acute lymphoblastic leukemia	11 (31.4)
	Acute myeloid leukemia	11 (31.4)
	Lymphoma	8 (22.9)
	Multiple myeloma	2 (5.7)
	Aplastic anemia	1 (2.9)
	Myelofibrosis	1 (2.9)

Frequency of co-morbid conditions in included patients was shown in Table 2.

**Table 2:** Frequency of concurrent diseases in enrolled patients (n = 35).

**Comorbidity**	**Frequency (%)**
No comorbidity	27 (77.1)
Heart failure	4 (11.4)
Diabetes	1 (2.9)
Hyperthyroidism	1 (2.9)
Asthma	1 (2.9)
Peptic ulcer	1 (2.9)

Test dose was administered in all of the patients while monitoring vital signs was performed only in 3 (8.75%) transplanted patients. With regard to the dosing, 25 (71.43%) patients received appropriate doses complying with the guidelines whereas dosing in 10 (28.57%) cases was inappropriate, of them 5 (14.28%) cases received higher doses and 5 (14.28%) patients were administered doses lower than the recommonded dose. The period of amphotericin administration was in accordance with the guidelines. Amphotericin B was administered at doses ranging from 0.5 mg/kg/day to 1.5 mg/kg/day with the median of 1.02 mg/kg/day. Total administered dose of this agent had a median (range) of 50 mg (20-40 mg). Treatment duration with amphotericin B was varied from 1 to 67 days with mean ± SD (19.4 ± 14.4 days). Duration of amphotericin B treatment according to NCCN guidelines was appropriately scheduled for 21 (60%) patients. From 14 individuals with improper duration of treatment, 7 patients received amphotericin B longer than periods justified by guidelines.

From 35 patients, 5 (14.28%) patients expired due to underlying blood disorder and 1(2.86%) patient died from aspergillosis. Deteriorating renal function (increase in serum creatinine and BUN) was observed in 9 (25.71%) patients. Five (55.55%) of them received amikacin and vancomaycin simultaneously with amphotericin. Vancomycin and amikacin were administered concomitantly in 29 (60.42%) and 6 (12.5%) patients, respectively. Documentation of culture results during therapy was performed in all transplanted patients but not in other neutropenic individuals. Fever resolved in 23 (65.7%) patients receiving amphotericin B as an empirical therapy. In patients whose fever was not resolved, 6 (17.14%) patients had received the drug for a shorter time-period due to the occurrence of adverse effects in 3 (8.57%) patients and early discharge before fever resolution in 3 other patients. Electrolytes imbalances and infusion related reactions were the most common adverse effects seen with amphotericin. The frequency of all detected adverse events was shown in Table 3.

**Table 3:** Frequency and percentage of adverse drug reactions observed with amphotericin B

**Side effects**	**Type of side effects**	**Frequency (%)**
Infusion –related	Fever Chills Nausea Vomiting Headache	8 (22.8) 8 (22.8) 9 (25.7) 9 (25.7) 8 (22.8)
Cardiovascular	Hypotension	18 (51.4)
Gastrointestinal	Loss of appetite Diarrhea	16 (45.7) 5 (14.2)
Electrolyte disturbances	Hypokalemia Hypomagnesia	22 (62.8) 20(57.1)
Nervous system	Fatigue Dizziness Insomnia Peripheral neuropathy	4 (11.4) 1 (2.8) 13 (37.1) 3 (8.5)
Musculoskeletal	Pain	7(20)
Skin	Pruritus	2(5.7)

## Discussion

Antifungal agents are administered as empirical therapy in neutropenic patients. Empirical antifungal therapy is initiated for the patients with persistent fever unresponsive to broad-spectrum antibiotic therapy (9). The results of our study showed that although usage of amphotericin B was mostly in accordance with reliable guidelines, however, dosage and duration of treatment were not appropriate in some patients. As mentioned in the guidelines, the dosage of amphotericin B in empirical therapy of febrile neutropenic patient is 0.5 to 0.7 mg/kg/d (10) and the recommended duration of empirical antifungal therapy is two weeks in stabilized patients (2). According to the result of this study, the dosage and duration of amphotericin B administration were inconsistent with the protocols in %29 and %40 of cases, respectively. Unlike our findings, in a study conducted by Pablo *et al. *(11) that compared conventional amphotericin B with the liposomal formulation, they observed that all patients received appropriate dosage. Moreover, in another study by Jeon *et al. *(12) the dosage and duration of treatment of patients with amphotericin B were in accordance with the recommended guidelines. In several other studies the dosage and duration of treatment of amphotericin B were shown to be appropriate (13-16). Improper dosage and duration of therapy with amphotericin B can be classified into two categories of prescribing high or low total cumulative doses of amphotericin. In case of administration of higher doses or prolonged courses of treatment, patients are at higher risk of developing adverse drug events as well as the potential harm due to high levels of amphotericin. Conversely, frequency of breakthrough infection increased by prescribing lower dose and duration of therapy. Furthermore, fever and neutropenia were not resolved with lower dose and shorter duration of amphotericin B therapy as seen in six patients in the present study. Due to the importance of neutropenic fever management in patients with cancer, the related health care system professionals should be familiar with the appropriate dosage and duration of amphotericin B administration as outlined in the guidelines. The result of our study showed that the physicians were not aware of guidelines regarding rational use of amphotericin B. This could be achieved by the attendance of clinical pharmacists in oncology and hematology wards during administration of amphotericin B in special patients. Consequently, better outcome and less adverse effects may be observed in the management of febrile neutropenia. The disparancy in the result of this study compared with those in other countries can be described partly by the fact that clinical pharmacy is a novel profession in Iran and lots of hospitals lack trained pharmacists.

As mentioned in the guidelines, monitoring the patient›s vital signs should be done every fifteen minutes prior to initiating amphotericin B treatment during test dose and then blood pressure and temperature should be recorded every 2 h during the infusion (17). In our study the evaluation of vital signs was only performed in patients hospitalized in BMT ward. This could be explained by the presence of clinical pharmacist in this ward as a part of health care team. However, as cardiac arrhythmia and infusion –related reactions appear to be associated with dose and infusion rate, monitoring of vital signs is crucial (18). Clinical pharmacists can arrange joint meetings with health care providers and explain the significance of monitoring patients during infusion of amphotericin B. Confirmed by several studies, presence of clinical pharmacy services in hospitals can further make the usage of drugs more rational and in compliance with the guidelines (19, 20).

Renal dysfunction is a frequent adverse drug phenomenon in patients receiving amphotericin B. The incidence of mild to moderate nephrotoxicity as a result of amphotericin B administration is approximately 50%, but the rate of severe toxicity is low (8%) and occurred when other nephrotoxic drugs administered concomitantly (7). As monitoring of renal function was a routine task in the hematology oncology and BMT wards, the detection of renal dysfunction was rapidly taken place. In our study, amphotericin B was administered simultaneously with vancomycin and amikacin in 29 and 6 patients, respectively, however fortunately only five patients on these combinations developed renal dysfunction. Confirming our findings, other studies also showed drug interactions of ampotericin B in cancer patients receiving other nephrotoxic drugs including vancomycin, cisplatin, amikacin and furosemide at the same course of treatment (20, 21). As cancer patients are prone to drug-drug interactions due to poly-pharmacy, the identification of patients at risk for amphotericin B induced nephrotoxicity and implementing supportive managements should be considered.

Although empiric antifungal therapy for management of neutropenic fever in patients unresponsive to antibiotics should be considered because of their susceptibility to invasive fungal infections, however decisions made on continuation of antifungal treatment should be judged based on the documented results of obtained cultures (8, 22). Nevertheless, in our study documentation of culture result was only performed in BMT patients. The importance of obtaining cultures should be emphasized to be sure of antifungal therapy duration appropriateness. 

In conclusion, our findings revealed that usage of amphotericin B in the Hematology-Oncology and Stem Cell Transplantation wards of the study hospital was not fully appropriate and according to the guidelines. As the management of febrile neutropenia in cancer patients is a crucial issue, health care providers’ awareness of antifungal drugs administration in particular amphotericin B monitoring and dosing schedules seems to be essential. Attendance of a clinical pharmacist along with education of nursing staff and monitoring of parameters according to current guidelines and updated protocols can minimize adverse effects and improve treatment outcomes.
